# A Case of Osseous Tumor, with a Tooth Imbedded in the Part

**Published:** 1860-02

**Authors:** M. H. Andrews


### A CASE OF OSSIOUS TUMOR WITH A TOOTH IMBEDDED IN THE PART.

BY M. H. ANDREWS, M. D.

Miss Bachellor, æt 16, about two years since observed a small, hard tumor, on the right side of the lower jaw, opposite the second bicuspid. It was without pain, hard to the touch, and firmly attached to the bone. It grew slowly for the first year, but about the first of November, 1858, it commenced extending along the jaw in both directions. From that time it grew fast until the 20th of June last. I cut down on the inside of the cheek and cut off the tumor smooth with the jaw, cutting away all the diseased structure that I could get at without removing the teeth which were not at this time disturbed. The outer portion of the tumor was a smooth hard shell of bone, seemingly an expansion of the jaw and alveolus, the inner portion soft gelatinous, or fatty, with small scales of bony matter diffused through the whole. The wound healed kindly and for about six weeks seemed to be doing well, but it soon showed that it was growing again, which it continued to do until the 13th of December, when assisted by Dr. J. Mansfield, of Niles, the tumor was again removed. It did not extend as far back on the jaw as before, but was developed forward nearly to the chin. This time all the teeth were extracted, the diseased portion removed as far as possible, and the surfaces cauterized with a hot iron.

On extracting the teeth the first bicuspid was found wanting, but on cutting out the diseased portion that extended under the teeth, the tooth was lying horizontally in the jaw with the crown towards the angle, the fang pointing forward; it occupied a position nearly under the molar teeth. It is now four weeks since the last operation, and there is no appearance of an unhealthy growth. It is healing kindly and everything seems to be doing well. The general health of the patient is good, seldom having any sick turns; has a good appetite and every way seems well.

The question arises, was the tooth the cause of the tumor, or a simple accidental circumstance.

